# Comparison study on statistical features of predicted secondary structures for protein structural class prediction: From content to position

**DOI:** 10.1186/1471-2105-14-152

**Published:** 2013-05-04

**Authors:** Qi Dai, Yan Li, Xiaoqing Liu, Yuhua Yao, Yunjie Cao, Pingan He

**Affiliations:** 1 College of Life Sciences, Zhejiang Sci-Tech University, Hangzhou, 310018, China; 2 College of Science, Hangzhou Dianzi University, Hangzhou, 310018, China; 3 College of Science, Zhejiang Sci-Tech University, Hangzhou, 310018, China

## Abstract

**Background:**

Many content-based statistical features of secondary structural elements (CBF-PSSEs) have been proposed and achieved promising results in protein structural class prediction, but until now position distribution of the successive occurrences of an element in predicted secondary structure sequences hasn’t been used. It is necessary to extract some appropriate position-based features of the secondary structural elements for prediction task.

**Results:**

We proposed some position-based features of predicted secondary structural elements (PBF-PSSEs) and assessed their intrinsic ability relative to the available CBF-PSSEs, which not only offers a systematic and quantitative experimental assessment of these statistical features, but also naturally complements the available comparison of the CBF-PSSEs. We also analyzed the performance of the CBF-PSSEs combined with the PBF-PSSE and further constructed a new combined feature set, PBF11CBF-PSSE. Based on these experiments, novel valuable guidelines for the use of PBF-PSSEs and CBF-PSSEs were obtained.

**Conclusions:**

PBF-PSSEs and CBF-PSSEs have a compelling impact on protein structural class prediction. When combining with the PBF-PSSE, most of the CBF-PSSEs get a great improvement over the prediction accuracies, so the PBF-PSSEs and the CBF-PSSEs have to work closely so as to make significant and complementary contributions to protein structural class prediction. Besides, the proposed PBF-PSSE’s performance is extremely sensitive to the choice of parameter *k*. In summary, our quantitative analysis verifies that exploring the position information of predicted secondary structural elements is a promising way to improve the abilities of protein structural class prediction.

## Background

Functionalities of proteins have been commonly believed to be determined by their unique 3-dimensional structures, which are determined by the exact spatial position of each atom [1]. In 1976, Levitt and Chothia studied the polypeptide chain topologies in a dataset of 31 globular proteins and proposed the concept of protein structural classes [2]. Proteins can be first classified into several structural folding classes, based on the type, amount, and spatial arrangement of their amino acid residues into potential secondary structure elements. SCOP (Structural Classification of Proteins) [3,4] and CATH (Class, Architecture, Topology and Homologous superfamily) [5,6] are two excellent protein structure databases that provide hierarchical structural classifications of proteins. The former database relies on a manual process to classify the structures, while the latter applies a combination of automated and manual procedures. There are 110,800 protein domains with known structural classes in SCOP database, and about 90% of them belong to the four major classes: all-α, all-*β*, *α+β* and *α*/*β* classes [3,4]. The two former classes include structures dominated by α-helices and β-strands, respectively. The two latter classes correspond to structures that include both helices and strands where in the case of the *α+β* class these secondary structures are segregated, whereas for *α*/*β* class the structures are interspersed.

The structural class has become one of the most important features for characterizing the overall folding type of a protein and played an important role in protein function analysis, prediction of protein folding rates, prediction of DNA-binding sites, protein fold recognition, reduction of the conformation search space, and implementation of a heuristic approach to find tertiary structure [7-12]. Due to the exponential growth of the number of known protein sequences, the burden of experimental screening methods regarding time and cost to find the 3-dimensional structure would become even more unbearable. If one can develop fast computational methods to predict at least some important characteristics of protein structures, which will help to speed up and reduce the cost for protein annotation. Therefore, computational methods are actively pursued to overcome the limitations of experimental screening methods.

Due to the importance of protein structural class prediction, various significant efforts have been devoted to this problem during the past 30 years, aiming to find a prediction model that automatically determine the structural class based on the protein sequences and predicted secondary structures [9,13-15]. Previous studies have shown that the protein structural class is strongly correlated with amino acid (AA) sequence, and the protein structural class can be predicted based on sequence-based features (SEFs) that are directly computed from AA sequences, such as the frequency of each AA in given proteins. These simple features are typically efficient, but they ignore the sequential order of AAs and the relationships among the distant AAs. To overcome these problems, high order SEFs have been proposed, such as composition of short polypeptides [16,17], pseudo AA composition [18], collocation of AA, function domain composition [19], and positions specific scoring matrices profiles computed by position specific iterative basic local alignment search tool (PSIBlast) [20]. However, these methods appear to be less effective in low-homology datasets whose average pair-wise sequence identities less than 40%. For instance, the reported overall accuracy for the widely used dataset 25PDB whose sequence homology is about 25%, were about 60% only [21,22].

In order to improve the prediction accuracy of low-similarity proteins, several new features of predicted secondary structures have been proposed [23-27]. Conveniently, we denote them by structure-based features (STFs). They exploit the fact that proteins with low sequence similarity but in the same structural class are likely to have high similarity in their corresponding secondary structure elements. Taking the above fact into account, Kurgan et al. computed the content of predicted secondary structural elements (*content*_*SE*_), normalized count of segments (NCount), length of the longest segment (MaxSeg), normalized length of the longest segment (NMaxSeg), average length of the segment (AvgSeg), normalized average length of the segment (NAvgSeg) based on the predicted secondary structures in protein structural class prediction [23]. Zheng and Kurgan counted the 3PATTERN of the predicted secondary structures to improve the *β*-turns prediction [24]. In MODAS, the predicted secondary structure information is employed to perform the prediction with evolutionary profiles [25]. In 2010, Liu and Jia found that *α*-helices and *β*-strands alternate more frequently in α/*β* proteins than in *α+β*proteins, and counted their alternating frequency as well as the content of parallel *β*-sheets and anti-parallel *β*-sheets [26]. Zhang et al. computed the transition probability matrix (TPM) of the reduced predicted secondary structural sequences and added it to protein structural class prediction [27]. With help of these STFs, the prediction accuracy has been improved significantly, between 80% and 85% on several low-similarity benchmark data-sets.

Despite the success of these STFs, they still focus mostly on the content of predicted secondary structure elements, and therefore to sometimes are unaware of the useful position-based information of elements in predicted secondary structures. The main goal of our research is to explore a potential way to capture the position information of predicted secondary structures and improve the prediction accuracy for such low-similarity data sets. In particular, we focus our investigation on the performance of the position-based features of the predicted secondary structure elements (PBF-PSSE) by comparing or combining with the content-based features of the predicted secondary structure elements (CBF-PSSE) in protein structural class prediction. The major content of this paper includes the following:

1. We presented a scheme to describe position of the predicted secondary structure elements and analyzed their distribution in all-*α*, all-*β*, *α*+*β* and *α*/*β* classes.

2. In order to numerically characterize the position information of secondary structures, we regarded the distance between two successive occurrences of an element as a variable and calculated its coefficient of the variability. This approach appears to be sensitive to the order of the structure elements because it is on the basis of all the distances between two successive occurrences of the elements.

3. We implemented a multi-class support vector machine (SVM) to predict protein structural class using PBF-PSSE, CBF-PSSE and both on four different benchmark datasets. Through a comprehensive comparison, we wanted to address the following questions with the aid of the well-known statistical indexes: (A) how well PBF-PSSE performs compared with the available CBF-PSSEs; (B) whether the CBF-PSSEs achieve a great improvement over the prediction accuracy when combining with the PBF-PSSE; (C) how well the proposed combined feature set, PBF11CBF-PSSE, performs in comparison with the available competing methods; (D) whether the PBF-PSSE’s ability depends on the maximal interval distance *k*.

## Methods

### Datasets

In order to facilitate comparison with previous studies, we selected four widely used low-homology benchmark datasets in which any pair of sequences shares twilight-zone similarity [22-27]. This means that any test sequence shares twilight-zone identity with any sequence in the training set used to generate the proposed classification model. The dataset, referred to as 25PDB, was selected using 25% PDBSELECT list [28], which includes proteins from PDB that were scanned with high resolution, and with low, on average about 25%, identity. The dataset was originally published in [22] and was used to benchmark two structural class prediction methods [29,30]. It contains 1673 proteins and domains. The secondary dataset, referred to as 1189, are downloaded from RCSB Protein Data Bank with the PDB IDs listed in the paper [22]. It contains 1092 proteins with 40% sequence identity. The third protein dataset, referred to as 640, was first studied in Chen et al. (2008) [20]. It contains 640 proteins with 25% sequence identity, and their classification labels are retrieved from the database SCOP [4]. The final dataset, named FC699, includes 858 sequences that share low 40% identity with each other. More details are presented in Table 1.

**Table 1:** Number of proteins belonging to different structural classes in the datasets

**Dataset**	**All-α**	**All-β**	**α/β**	**α+β**	**Total**
25PDB	443	443	346	441	1673
640	138	154	177	171	640
FC699	130	269	377	82	858
1189	223	294	334	241	1092

### Protein secondary structure prediction

Every amino acid in a protein sequence can be predicted into one of the three secondary structural elements H (helix), E (strand), and C (coil). It is a problem known as protein secondary structure prediction, and many computational approaches have been developed in the past decades to predict the 3-state secondary structure from protein sequences. In this study, PSIPRED [31] was chosen to predict protein secondary structure because it outperforms other competing prediction methods [32,33]. If you want to obtain the prediction secondary structure of protein 1PET whose amino acid sequence is DSITYRVRKGDSLSSIAKRHGVNIKDVMRWNSDTANL QPGDKLTLFVK, you can submit it to PSIPRED and obtain the predicted secondary structure like this CCEEEEECCCCCHHHHHHHHCCCCCCCCCCCCCCEEEEEEC. The available structure-based predictions take the predicted secondary structure sequence as input, but they are not tied to any specific tool for the secondary structure prediction. Any improved secondary structure prediction would generally lead to a high accuracy structure-based protein structural class prediction method [34-36].

### Content-based features of predicted secondary structure elements (CBF-PSSE)

Prediction methods, using the protein SEFs, achieve promising results in protein structural class prediction, unfortunately the accuracy is limited. Some studies indicate that the contents and spatial arrangements of secondary structural elements are also significant factors that influence the protein intricate functions or structures [23-27], so various CBF-PSSEs have been proposed, such as the content of the predicted secondary structure elements or segments. Since this paper focuses on comparison study on statistical features of predicted secondary structures, we first reviewed the available CBF-PSSEs with better performance in protein structural class predication.

1. Predicted secondary structure elements’ content (*content*_*SE*_)

Predicted secondary structure elements’ content, denoted by *content*_*SE*_, is one of the most widely used CBF-PSSEs [23,25-27]. It can be calculated by taking a sliding window and scanning through the predicted secondary structure sequences

(1) $$\mathrm{conten}t_{\mathrm{SE}}=\frac{\mathrm{Coun}t_{\mathrm{SE}}}{\sum_{\chi \in \left\{C,H,E\right\}}\mathrm{Coun}t_{x}},$$

where *Count*_*SE*_ is the total number of occurrence of the predicted secondary structure element *SE*, *SE* ∈ {*C*, *H*, *E*}. *H*, *E* and *C* denote *α*-helix, *β*-strand and coil, respectively.

2. First and second order composition moment vector (*CMV*) [23,25-27], another important CBF-PSSE, can be calculated as follows:

(2) $$\mathrm{CM}V_{\mathrm{SE}}^{k}=\frac{\sum_{j=1}^{\mathrm{Coun}t_{\mathrm{SE}}}PO_{\mathrm{SEj}}^{k}}{\prod_{d=1}^{k}\left(B-d\right)},$$

Where *PO*_*SEj*_ represents the *j*th position of the predicted secondary structure element *SE*, *N* is the length of the predicted secondary structure sequence, and *k* is the order of the composition moment vector.

3. There are many different arrangements of *α*-helices and *β*-strands among four main classes. In order to distinguish these arrangements, the longest segment, average length of the segments and their normalized forms have been proposed and calculated as follows:Length of the longest segment (*MaxSeg*_*SE*_) [23,25-27]

(3) $$\mathrm{MaxSe}g_{\mathrm{SE}}=\mathrm{MaxLen}\left(\mathrm{SEG}:\mathrm{SE}G_{\mathrm{SE}}\right),$$

where *MaxLen* is the maximal function of segment length, and *SEG*_*SE*_ is the segments composed of structure element *SE*.

4. Normalized length of the longest segment (*NMaxSeg*_*SE*_) [23,25-27]

(4) $$\mathrm{NMaxSe}g_{\mathrm{SE}}=\frac{\mathrm{MaxLen}\left(\mathrm{SEG}:\mathrm{SE}G_{\mathrm{SE}}\right)}{N},$$

where *N* is the length of the predicted secondary structure sequence.

5. Average length of the segment (*AvgSeg*_*SE*_) [23,25-27]

(5) $$\mathrm{AvgSe}g_{\mathrm{SE}}=\frac{\sum \mathrm{Len}\left(\mathrm{SEG}:\mathrm{SE}G_{\mathrm{SE}}\right)}{\mathrm{Conten}t_{\mathrm{SE}G_{\mathrm{SE}}}},$$

where *Len* is the function of segment length, and $\mathrm{Conten}t_{\mathrm{SE}G_{\mathrm{SE}}}$ denotes the total appearances of the *SEG*_*SE*_.

6. Normalized average length of the segment (*NAvgSeg*_*SE*_) [23,25-27]

(6) $$\mathrm{NAvgSe}g_{\mathrm{SE}}=\frac{\sum \mathrm{Len}\left(\mathrm{SEG}:\mathrm{SE}G_{\mathrm{SE}}\right)}{\mathrm{Conten}t_{\mathrm{SE}G_{\mathrm{SE}}}},$$

where *N* is the length of the predicted secondary structure sequence.

7. 3PATTERN

Zheng and Kurgan proposed 3PATTERN method and enhanced the prediction accuracy of *β*-turns to over 80% based on the predicted secondary structure sequences [24]. 3PATTERN_*m, k*_ denotes a specific configuration of the secondary structure for the central and the two adjacent residues, where *m* is the pattern type. For *m* = 1 and *k* = C, the secondary structure prediction would be CCC, and for *m* = 2, 3, and 4 the prediction would be CCx, xCC, and xCx, respectively, where *x* ∈ {*EH*}. They encode whether the central (predicted) residue is located inside a secondary structure segment or at the interface between two segments.

8. Alternating frequency of *α*-helices and *β*-strands and proportion of parallel *β*-sheets and anti-parallel *β*-sheets (*APPA*)

In 2010, Liu and Jia found that the *α*-helices and the *β*-strands alternate more frequently in *α*/*β* proteins than in *α*+*β* proteins, so they counted the alternating frequency as well as the content of the parallel *β*-sheets and the anti-parallel *β*-sheets [26]. The normalized alternating frequency of the *α*-helices and the *β*-strands (Altn/N) is defined as follows:

(7) $$\mathrm{NAl}t_{\mathrm{SE}}=\frac{\mathrm{Conten}t_{\alpha -\beta }}{\mathrm{SeqLen}},$$

where *Content*_*α-β*_ is the total alternation of *α*-helices and *β*-strands, and *SeqLen* is the length of the predicted secondary structure sequence.

9. The transition probability matrix of the reduced segment sequence (*TPM*)

In 2010, Zhang *et al.* ignored coil segments and transformed a secondary structure sequence into a segment sequence that is only composed of helix segments and strand segments [27]. They defined transition probability matrix (*TPM*) of the reduced segment sequence as follows:

(8) $$\mathrm{TPM}=\left(\begin{array}{cc} P_{\alpha \alpha } & P_{\alpha \beta }\\ P_{\beta \alpha } & P_{\beta \beta } \end{array}\right),$$

where

Pαiαj=Contentαiαj∑t=12Contentαiαj∑t=12Contentαiαj≠00∑t=12Contentαiαj=0

*a*_*i*_ represents the *i*th element of the state space {*α, β*}, and *Content*_*aiaj*_ is total appearance of the incident, *a*_*i*_ is followed by letter *a*_*j*_ in the segment sequence.

### Representation of the secondary structure elements’ position

The above CBF-PSSEs focus mainly on the content of predicted secondary structure elements, and therefore they will ignore the useful position distribution of elements in predicted secondary structures. For example, given a predicted secondary structure sequence CCEEEEECCCCCHHHHHHH, if we move its last seven HHHHHHH to the third position of the structure sequence, we will get another secondary structure sequence CCHHHHHHHEEEEECCCCC according to the elements’ position, but the elements’ content does not change. So when assigning the protein structural classes, the secondary structure elements’ position should be considered as another deciding factor. Instead of counting the occurrences of distinct helix, strand and coil segments, this paper analyzed the distribution of the successive occurrences of a predicted secondary structure element.

To find all occurrences of an element δ in the predicted secondary structure sequence *s*, the random indicator *ϕ*_*i*_(*δ*) is defined as follows:

$$\phi_{i}\left(\delta \right)=\left\{\begin{array}{cc} i & \mathrm{if}\;s_{i}=\delta \\ 0 & \mathrm{otherwise} \end{array}\right)$$

With help of the random indicator, we transformed a predicted secondary structure sequence into three position sequences. After removing zeros from the position sequences, we obtained three numerical sequences denoted as *Po*(δ). Take the above sequence *s*=CCHHHHEEEEECCCCCHHH as an example, its numerical sequences *Po*(*C*), *Po*(*H*) and *Po*(*E*) are:

$$\begin{array}{l} \mathrm{Po}\left(C\right)=\left(1,2,12,13,14,15,16\right),\\ \mathrm{Po}\left(H\right)=\left(3,4,5,6,14,15,16,17,18,19\right),\\ \mathrm{Po}\left(E\right)=\left(7,8,9,10,11\right). \end{array}$$

From the numerical sequence *Po*(δ), it is easily to deduce that whether two successive occurrences of the element *δ* belong to the same helix (strand and coil) or not. If the interval distance between two successive occurrences of the element δ, referred to as *Dis*(δ), is equal to 1, they will form a helix (strand and coil), otherwise they belong to different helixes (strands and coils). Based on the numerical sequence *Po*(δ), we computed the interval distances between two successive occurrences of the element δ and got a novel numerical characteristic sequence denoted by *N*(δ). Take the above position sequences as an example, their numerical characteristic sequences *N* (δ) are:

$$\begin{array}{l} N\left(C\right)=\left(1,10,1,1,1,1\right),\\ N\left(H\right)=\left(1,1,1,8,1,1,1,1,1\right),\\ N\left(E\right)=\left(1,1,1,1\right). \end{array}$$

These numerical sequences *N*(δ) not only indicate the structure elements’ content, but also reflect distribution information of the interval distances between their consecutive occurrences.

### Position-based feature of predicted secondary structure elements (PBF-PSSE)

**Figure 1:**
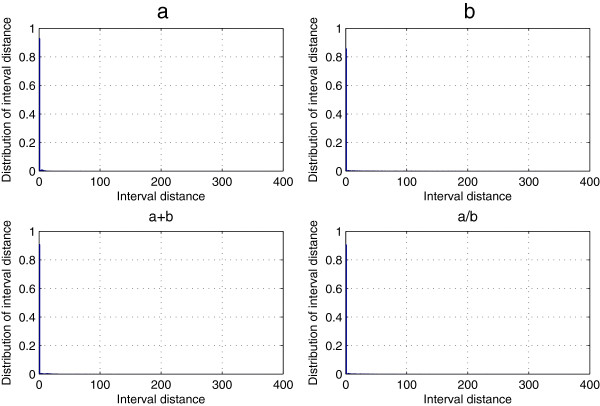
**Distribution of the interval distance *Dis*(*H*) for the 25PDB dataset.** Distribution (*Dis*(*H*)) of the interval distance between two nearest structure elements H for the 25PDB dataset, **a**, **b**, a+b and a/b denotes all-α, all-*β*, *α*+*β* and *α* / *β* classes.

**Figure 2:**
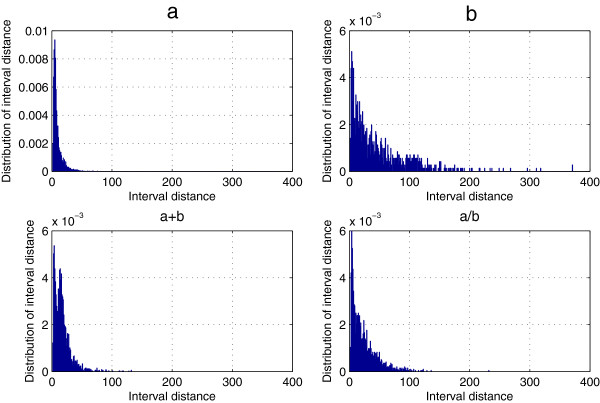
**Distribution of the interval distance *Dis*(*H*) (*Dis*(*H*) >1) for the 25PDB dataset.** Distribution (*Dis*(*H*)) of the interval distance between two nearest structure elements H for the 25PDB dataset. Here, *Dis*(*H*) >1, and **a**, **b**, a+b and a/b denotes all-*α*, all-*β*, *α* + *β* and *α* / *β* classes.

Given a structure element δ, we can transform a predicted secondary structure sequence into a numerical characteristic sequence *N*(δ) that provides a new profile of the correlation structure of the given structure sequence. Here, we chose 25PDB dataset that includes 443 all-*α*, 443 all-*β*, 346 *α*/*β*, and 441 *α*+*β* proteins. Using the random indicator *ϕ*(*H*) and statistical method, we obtained 1673 numerical characteristic sequences *N*(*H*) and calculated the count of the interval distance *Dis*(*H*) for all-*α*, all-*β*, *α*/*β* and *α*+*β* classes, which is represented in Figure 1. It is easy to find that more than 80% of *Dis*(*H*) is equal to 1 among all-*α*, all-*β*, *α*/*β*, and *α*+*β* classes, and the rest are too small. Figure 2 shows distribution of *Dis*(*H*) >1 more clearly because *Dis*(*H*) =1 has been omitted. Take a closer look at Figure 2, we found that the count of *Dis*(*H*) >1 in the all-*α* class is larger than the other classes, which is coincident with the fact that the all-*α* class is dominated by *α*-helices. Also, the distribution of *Dis*(*H*) >1 is more concentrative in the *α*/*β* class and the *α*+*β* class than that in the all-*β*class.

Since *Dis*(δ) varies with different predicted secondary structure sequences, it can be regarded as a discrete random variable. Given a random variable *Dis*(δ), and a positive integer *n* , *p*(*Dis*(δ)=*n*) is the probability that *Dis*(δ) takes the value *n*. The collection of pairs (*Dis*(δ)=*n*, *P*(*Dis*(δ)=*n*)), for all positive integer *n*, is the probability distribution of the *Dis*(δ) listed in Table 2.

**Table 2:** **Probability distribution of the *Dis(δ)***

					
*Dis(δ)*	*Dis*(*δ*) = 1	*Dis*(*δ*)=2	…	*Dis*(*δ*)=*n*	…
*P*	*P*(*Dis(δ)*=1)	*P*(*Dis*(*δ*) = 2)	…	*P*(*Dis(δ)*=*n*)	…

Based on above distribution function, we calculated two numerical characteristics: semi-mean *Semi-E*_(*k*)_(*δ*) and semi-variance *Semi-D*_(*k*)_(*δ*) defined by:

(9) $$\mathrm{Semi}-E_{\left(k\right)}\left(\delta \right)=\sum_{\mathrm{Dis}\left(\delta \right)=1}^{k}\mathrm{Dis}\left(\delta \right)\times P\left(\mathrm{Dis}\left(\delta \right)\right),$$

(10) $$\begin{array}{lll} \mathrm{Semi}-D_{\left(k\right)}\left(\delta \right) & = & \sum_{\mathrm{Dis}\left(\delta \right)=1}^{k}{\left(\mathrm{Dis}\left(\delta \right)\right)}^{2}\times P\left(\mathrm{Dis}\left(\delta \right)\right)\\ & - & {\left[\sum_{\mathrm{Dis}\left(\delta \right)=1}^{k}\mathrm{Dis}\left(\delta \right)\times P\left(\mathrm{Dis}\left(\delta \right)\right)\right]}^{2}. \end{array}$$

Here, *Semi-E*_(*k*)_(*δ*)and *Semi-D*_(*k*)_(*δ*) are not mean and variance because we only added the former *k* values rather than all the parameter values. The PBF-PSSE *C*_(*k*)_(*δ*) is then defined as the ratio of the standard *Semi-D*_(*k*)_ to *Sime-E*(*k*)

(11) $$C_{\left(k\right)}\left(\delta \right)=\frac{\mathrm{Semi}-E_{\left(k\right)}\left(\delta \right)}{\sqrt{\mathrm{Semi}-D_{\left(k\right)}\left(\delta \right)}}$$

*C*_(*k*)_(*δ*) is the reciprocal of coefficient of variation which shows the extent of variability in relation to mean of the population. For the convenience of comparison, we denoted *C*_(*k*)_(*δ*) based on all the parameter values as *C*_(*F*)_(*δ*).

In probability theory and statistics, the coefficient of variation is a normalized measure of dispersion of a probability distribution. It is also known as unitized risk or the variation coefficient. The coefficient of variation is also common in applied probability fields such as renewal theory, queuing theory, and reliability theory. The coefficient of variation is useful because the standard deviation of data must always be understood in the context of the mean of the data. Instead, the actual value of the coefficient of variation is independent of the unit in which the measurement has been taken, so it is a dimensionless number. For comparison between data sets with different units or widely different means, one should use the coefficient of variation instead of the standard deviation. Here, *C*_(*k*)_(*δ*) is used to describe the position distribution of predicted secondary structure elements.

### Prediction assessment

In this paper, we adopted Vapnik’s support vector machine to predict the protein structural class [37]. Support vector machine is one type of learning machine based on statistical learning theory. Since there are four structural classes, we chose the multi-class prediction method for protein structural class prediction. Given a test protein of unknown category, the SVM first maps the input vectors into one feature space (perhaps with a higher dimension). Then within the space mentioned above, it finds an optimized linear division to solve two-class or multi-class problem [38]. Finally, a prediction label to the test sample is assigned according to this way. A more detailed description of SVM is in Vapnik’s book [37].

Among the three kinds of cross-validation methods (the single-test-set analysis, sub-sampling and jackknife analysis), the jackknife test is supposed to be the most effective one [39]. Here, we used it to evaluate the performance of the proposed method. We also considered standard performance measures over structural class, including the accuracy for class *C*_*j*_ and overall accuracy, which was defined as the fraction of class *C*_*j*_ or all the proteins tested that are classified correctly.

(12) $$\mathrm{Accurac}y_{j}=\frac{TP_{j}}{\left|C_{j}\right|},$$

(13) $$\mathrm{Overall}\;\mathrm{accuracy}=\frac{\Sigma_{j}TP_{j}}{\Sigma_{j}\left|C_{j}\right|},$$

where *TP*_*j*_ is the number of true positives, and |*C*_*j*_| is the number of proteins in each structural class *C*_*j*_ (all-*α*, all-*β*, *α*/*β* and *α*+*β* classes).

### Selection of parameters C and gamma

We selected the Gaussian as the kernel function for the SVM because its superiority for solving nonlinear problems compared with other kernel functions [40]. Here, we selected the parameters for the sake of getting the highest overall prediction as possible. Then a simple grid search strategy over C and gamma values based on 10-fold cross-validation for each dataset was selected, where C and gamma were allowed to take the values only between 2^-5^ to 2^5^.

## Results and discussion

This section includes discussion of the selected feature, experiment results, comparison of PBF-PSSE, CBF-PSSE, and the proposed combined feature set on four benchmarking datasets. In the first step, we used the PSIPRED to predict the secondary structures of protein. Then, the representation was employed to represent a predicted secondary structure as three numerical sequences, from which we calculated the PBF-PSSE, a 3-feature set. Finally, the PBF-PSSE, CBF-PSSE and the proposed combined feature set were fed into support vector machine to make prediction of its protein structural class, respectively. We reported overall accuracy and accuracy for each structural class.

### Prediction accuracy of PBF-PSSE *C*_*F*_(*δ*) for four benchmark datasets

Four widely used datasets with low sequence identity were used in this study, including 25PDB that comprises 1673 proteins of about 25% sequence identity, 640 that includes 640 proteins of about 25% sequence identity, FC699 with 858 proteins of about 40% sequence identity, and 1189 that contains 1092 proteins of about 40% sequence identity. The results obtained by the PBF-PSSE *C*_*F*_(*δ*) were shown in Table 3. Table 3 shows that the overall accuracies obtained by the PBF-PSSE *C*_*F*_(*δ*) are 75.25%, 79.8%, 85.7% and 78.4% for the 25PDB, 640, FC699 and 1189 datasets, respectively.

**Table 3:** Prediction accuracy of CBF-PSSEs and PBF-PSSE for four datasets

**Dataset**	**Type**	**Method**	**Prediction accuracy (%)**				
			**All-α**	**All-β**	**α/β**	**α+β**	**Overall**
25PDB	CBF-PSSE	*content*_*SE*_	89.39	76.75	68.21	60.54	74.06
		CMV	88.04	69.53	66.76	62.81	72.09
		*MaxSeg*_*SE*_	83.30	68.62	58.67	56.24	67.18
		*NMaxSeg*_*SE*_	79.68	70.65	70.81	70.98	73.16
		*AvgSeg*_*SE*_	79.91	69.53	68.79	70.75	72.44
		*NAvgSeg*_*SE*_	35.44	96.61	0	6.12	36.58
		3PATTERN	76.75	66.37	71.68	57.14	67.78
		APPA	64.56	62.08	55.49	57.37	60.13
		TPM	83.52	73.14	72.54	56.01	71.25
	PBF-PSSE		**74.72**	**77.88**	**69.08**	**78.00**	**75.25**
640	CBF-PSSE	*content*_*SE*_	89.86	77.27	81.36	64.33	77.66
		CMV	84.78	76.62	88.14	57.89	76.56
		*MaxSeg*_*SE*_	73.91	70.13	74.58	52.05	67.34
		*NMaxSeg*_*SE*_	79.71	75.32	85.31	60.82	75.16
		*AvgSeg*_*SE*_	79.71	66.88	88.70	60.23	73.91
		*NAvgSeg*_*SE*_	8.70	0	100	0	29.53
		3PATTERN	65.94	60.39	87.01	52.05	66.72
		APPA	63.04	64.29	65.54	53.22	61.41
		TPM	76.81	68.83	84.75	62.57	73.28
	PBF-PSSE	*C*_*F*_(*δ*)	**76.09**	**78.57**	**84.75**	**78.95**	**79.84**
FC699	CBF-PSSE	*content*_*SE*_	84.62	91.45	93.9	34.15	86.01
		CMV	82.31	90.33	94.16	21.95	84.27
		*MaxSeg*_*SE*_	83.85	86.25	97.08	12.2	83.57
		*NMaxSeg*_*SE*_	83.08	86.62	92.84	51.22	85.43
		*AvgSeg*_*SE*_	86.15	85.87	94.16	46.34	85.78
		*NAvgSeg*_*SE*_	3.85	0	99.73	0	44.41
		3PATTERN	76.92	80.3	95.76	50	83.68
		APPA	63.85	75.09	95.49	0	75.17
		TPM	90	88.48	87	51.22	84.5
	PBF-PSSE	*C*_*F*_(*δ*)	**88.46**	**81.41**	**88.86**	**80.49**	**85.66**
1189	CBF-PSSE	*content*_*SE*_	86.1	83.67	84.43	55.19	78.11
		CMV	83.41	81.63	84.13	36.93	72.89
		*MaxSeg*_*SE*_	82.96	80.95	72.75	41.49	70.15
		*NMaxSeg*_*SE*_	79.82	80.61	81.74	53.94	74.91
		*AvgSeg*_*SE*_	78.48	73.47	82.93	48.55	71.87
		*NAvgSeg*_*SE*_	0	0	1	0	30.59
		3PATTERN	65.92	70.07	83.53	44.81	67.77
		APPA	61.88	68.37	76.95	31.54	61.54
		TPM	85.2	78.23	76.05	56.02	74.08
	PBF-PSSE	*C*_*F*_(*δ*)	**81.61**	**82.31**	**79.94**	**68.46**	**78.39**

The accuracy of each class and overall accuracy obtained by CBF-PSSEs and PBF-PSSE for datasets 25PDB, 640, FC699 and 1189.

Among the four structural classes, *α*+*β* is the most hardest to predict. Its average accuracy is always about 5-10% lower than the other three structural classes [22]. But in the PBF-PSSE *C*_*F*_(*δ*), the average accuracy for the *α*+*β* class is 81.76%, which is 0.63-20.21% higher than the other three structural classes. These results hence clearly indicate that the PBF-PSSE *C*_*F*_(*δ*) is more suitable to characterize the helix’s and strand’s distribution.

### Comparison between PBF-PSSE *C*_*F*_(*δ*) and CBF-PSSEs

PBF-PSSE *C*_*F*_(*δ*) aims at the structure elements’ position distribution among all-α, all-*β*, *α*/*β* and *α*+*β* classes. For a better understanding of the PBF-PSSE *C*_*F*_(*δ*), a comparison with other statistical features was performed. Since this paper focuses on comparison study on statistical features of predicted secondary structures, we compared PBF-PSSE *C*_*F*_(*δ*) with nine available CBF-PSSEs on the same data sets. In this section, we selected the accuracy of each class and overall accuracy as evaluation methods, which are summarized in Table 3.

In the 25PDB experiment, PBF-PSSE *C*_*F*_(*δ*) performs better than all CBF-PSSEs, with overall accuracy 75.25%. Among all the CBF-PSSEs, *content*_*SE*_ is significantly better than all other CBF-PSSEs, and the next best CBF-PSSE is *NMaxSeg*_*SE*_. In the 640 experiment, the PBF-PSSE *C*_*F*_(*δ*) achieves the highest overall prediction accuracy among all the PBF-PSSE and the CBF-PSSEs. Among the CBF-PSSEs, *content*_*SE*_ is better than all other CBF-PSSEs, and the next best one is *NMaxSeg*_*SE*_. In the FC699 experiment, two CBF-PSSEs, *content*_*SE*_ and *AvgSeg*_*SE*_, outperform the PBF-PSSE *C*_*F*_(*δ*). As for the dataset 1189, the PBF-PSSE *C*_*F*_(*δ*) is better than all the CBF-PSSEs, with overall accuracy 78.39%. The next best one is *content*_*SE*_, and the other features lag behind.

As for *α*+*β* class, the accuracies of the PBF-PSSE *C*_*F*_(*δ*) for datasets 25PDB, 640, FC699 and 1189 are 78.00%, 78.95%, 80.49% and 68.46%, which are 7.02%, 14.62%, 29.27% and 12.44% higher than the best-performing CBF-PSSEs, respectively.

From the above experiments, we can see that both the PBF-PSSE *C*_*F*_(*δ*) and the CBF-PSSEs make their own positive contributions to the predictions. The PBF-PSSE *C*_*F*_(*δ*)performs better than CBF-PSSEs among three experiments, especially for *α*+*β* class prediction. *content*_*SE*_ achieves the best performance among all the CBF-PSSEs.

### Performance of the CBF-PSSE combined with the PBF-PSSE *C*_*F*_(*δ*)

PBF-PSSE and CBF-PSSEs are the two most important kind feature sets of predicted secondary structures for protein structural class prediction. It can be seen that when the features are used individually, the resulting overall prediction accuracy for four datasets is all well above 25%. It indicates that these predictions are unlikely to be random, since random assignment of protein classes generally leads to an accuracy value of about 25%. In other words, every feature subset makes its own positive contributions to the predictions.

The differences between the PBF-PSSE and the CBF-PSSEs are that the position information is considered in the former, and the content information is explored in the latter. For a better understanding of the PBF-PSSE *C*_*F*_(*δ*), we combined the PBF-PSSE *C*_*F*_(*δ*) with CBF-PSSEs to form some new combined feature sets. Through the experiments, we wanted to address how well the CBF-PSSEs perform when combining with the PBF-PSSE *C*_*F*_(*δ*).

Table 4 lists prediction accuracy obtained with the CBF-PSSEs combined with the PBF-PSSE *C*_*F*_(*δ*). From Table 3, we note that the PBF-PSSE *C*_*F*_(*δ*) provides the overall prediction accuracy that is only comparable to the CBF-PSSE *content*_*SE*_, and it even gives a lower accuracy values (85.66% v.s. 88.46%) for the datasets FC699. But when combining with the CBF-PSSE *content*_*SE*_, the prediction accuracy of the PBF-PSSE *C*_*F*_(*δ*) is improved by about 9.0%. Specifically, there are the accuracy improvements of 29.94%, 5.94%, 9.21%, and 6.04% for the datasets 25PDB, 640, FC699 and 1189, respectively. Table 4 shows that all the CBF-PSSEs’ prediction abilities are improved by combining with PBF-PSSE *C*_*F*_(*δ*), except for *MaxSeg*_*SE*_ and 3PATTERN. There are about 4.43%~48.28% higher than the prediction methods solely from the CBF-PSSEs.

**Table 4:** **The overall prediction accuracy for four data sets obtained by the CBF-PSSEs combined with the PBF-PSSE *C***_***F***_**( *δ *)**

**Methods**	**25PDB**	**640**	**FC699**	**1189**
*content*_*SE*_+*C*_*F*_(*δ*)	83.14	85.78	94.87	84.43
*CMV* +*C*_*F*_(*δ*)	81.83	84.38	90.91	82.88
*MaxSeg*_*SE*_+*C*_*F*_(*δ*)	68.56	67.34	83.57	70.05
*NMaxSeg*_*SE*_+*C*_*F*_(*δ*)	82.55	85.16	91.38	82.97
*AvgSeg*_*SE*_+*C*_*F*_(*δ*)	83.86	84.69	94.06	85.07
*NAvgSeg*_*SE*_+*C*_*F*_(*δ*)	75.55	79.84	85.55	78.39
3PATTERN +*C*_*F*_(*δ*)	67.78	66.72	83.68	67.77
*APPA* +*C*_*F*_(*δ*)	81.29	84.06	91.14	81.41
*TPM* +*C*_*F*_(*δ*)	82.37	84.22	92.89	80.59

**Figure 3:**
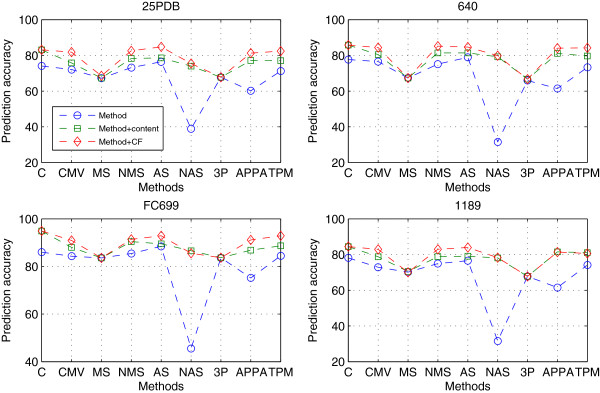
**Performance of the CBF-PSSEs combined with the PBF-PSSE *C***_***F***_**(*δ*) and the CBF-PSSE *content***_***SE***_**.** Performance of the CBF-PSSEs combined with the PBF-PSSE *C*_*F*_(*δ*) and the CBF-PSSE *content*_*SE*_, where C, MS, NMS, AS, NAS and 3P denote the *content*_*SE*_ , *MaxSeg*_*SE*_ , *NMaxSeg*_*SE*_, *AvgSeg*_*SE*_ , *NAvgSeg*_*SE*_ and 3PATTERN.

For comparison purpose, the CBF-PSSEs combined with the CBF-PSSE *content*_*SE*_ were also tested. Here, we chose the CBF-PSSE *content*_*SE*_ because it is one of the most efficient CBF-PSSEs and often combined with predicted secondary structures or protein sequence [23-27]. The comparison of the CBF-PSSEs combined with the PBF-PSSE *C*_*F*_(*δ*) and with the CBF-PSSE *content*_*SE*_ is presented in Figure 3, and more details can be found in Additional file 1: Table S1.

As would be expected, the prediction accuracy of the different combined feature sets shows two clear trends: (i) when exploring the PBF-PSSE *C*_*F*_(*δ*) and the CBF-PSSE *content*_*SE*_, all the CBF-PSSEs’ prediction abilities are improved except for *MaxSeg*_*SE*_ and 3PATTERN; (ii) it is interesting to note that high accuracy of prediction can be achieved by the CBF-PSSE combined with the PBF-PSSE *C*_*F*_(*δ*). These experiments further demonstrate that the PBF-PSSE *C*_*F*_(*δ*) plays an important role in recognition of protein structural classes and can be used to improve the prediction accuracy. PBF-PSSE and CBF-PSSE have to work closely so as to make significant and complementary contributions to protein structural class prediction.

### Comparison of the proposed PBF11CBF-PSSE with the competing predictions based on the predicted secondary structures

The above experiments show that the PBF-PSSE and the CBF-PSSE make significant and complementary contributions to protein structural class prediction, so this paper proposed a new combined feature set, denoted by PBF11CBF-PSSE, that consists of the PBF-PSSE *C*_*F*_(*δ*) and widely used 11-dimension CBF-PSSE set. Table 5 presents the accuracy of the proposed PBF11CBF-PSSE. To evaluate the efficiency of the PBF11CBF-PSSE, we compared it with the competing prediction methods on the same data sets. Since PBF11CBF-PSSE was constructed based on the information of the predicted secondary structure, the evaluated prediction methods should be based on predicted secondary structure information only. These competing methods include RKS-PPSC [41], Liu and Jia [26], Zhang *et al.*[27] and Ding *et al.*[42]*.* Table 5 lists the accuracy of each class and overall accuracy of all the evaluated prediction methods.

**Table 5:** Prediction accuracy of the proposed PBF11CBF-PSSE for four datasets and comparison with the competing prediction methods

**Dataset**	**Method**	**Prediction accuracy (%)**				
		**All-α**	**All-β**	**α/β**	**α+β**	**Overall**
25PDB	RKS-PPSC [42]	92.8	83.3	**85.8**	70.1	82.9
	Liu and Jia [26]	92.6	81.3	81.5	76.0	82.9
	Zhang et al. [27]	95.0	85.6	81.5	73.2	83.9
	Ding et al. [42]	95.03	81.26	83.24	77.55	84.34
	Proposed PBF11CBF-PSSE	**98.65**	**85.78**	79.19	**79.82**	**86.25**
640	RKS-PPSC [41]	89.1	**85.1**	88.1	71.4	83.1
	Ding et al. [42]	94.93	76.62	89.27	74.27	83.44
	Proposed PBF11CBF-PSSE	**97.1**	81.17	**89.27**	**79.53**	**86.41**
FC699	Liu and Jia [26]	97.7	88.0	89.1	**84.2**	89.6
	Proposed PBF11CBF-PSSE	**100**	**97.03**	**96.55**	73.17	**94.99**
1189	RKS-PPSC [41]	89.2	86.7	82.6	65.6	81.3
	Zhang et al. [27]	92.4	**87.4**	82.0	**71.0**	83.2
	Ding et al. [42]	93.72	84.01	83.53	66.39	81.96
	Proposed PBF11CBF-PSSE	**97.76**	86.39	**84.73**	70.54	**84.71**

The accuracy of each class and overall accuracy of the proposed PBF11CBF-PSSE for four datasets, and comparison with the competing prediction methods based on protein prediction secondary structures.

As for 25PDB dataset, the proposed PBF11CBF-PSSE outperforms all other methods. There are only two methods that provide the overall accuracy over 84%. One is PBF11CBF-PSSE, and the other is the method proposed by Ding *et al.*[42]. But the overall accuracy of PBF11CBF-PSSE is 86.25%, which is 1.91% higher than Ding’s method [42]. Results shown in Table 4, which concern on the 640, FC 699 and 1189 datasets, are consistent with the results on the 25PDB dataset. The overall accuracies yielded by PBF11CBF-PSSE for datasets 640, FC699 and 1189 are 86.41%, 94.99% and 84.71%, which are 2.97%, 5.39% and 1.51% higher than the existing best-performing method. We attribute higher overall accuracy to the PBF-PSSE *C*_*F*_(*δ*) involved in the PBF11CBF-PSSE.

In addition, we further compared the results of the proposed PBF11CBF-PSSE with two popular methods, MODAS [12] and SCPRED [23], in which the predicting sequence information was combined with evolutionary profiles or protein sequences to predict the protein structural classes. The overall accuracies yielded by MODAS for datasets 25PDB and 1189 are 81.4% and 83.5%, which are 4.85% and 1.21% lower than the proposed PBF11CBF-PSSE. As for SCPRED method, its overall accuracies for datasets 25PDB and FC699 are 79.7% and 87.5%, which are 6.55% and 7.49% lower than the proposed PBF11CBF-PSSE. These results also demonstrate that the position information from the predicted secondary structures could be more promising to improve protein structural class prediction because it is more suitable to represent the structure elements’ order information, certain local interactions and spacial arrangements of the *α*-helices and the *β*-strands.

### Influence of parameter *k* in the PBF-PSSE *C*_*F*_(*δ*)

PBF-PSSE *C*_(*k*)_(*δ*) is the reciprocal of coefficient of variation which shows the extent of variability in relation to mean of the population. It describes the position distribution of predicted secondary structure elements and contributes to the protein structural class prediction. However, it should be noted that *C*_(*k*)_(*δ*) relies heavily on the *k* parameter, the given interval distance.

**Figure 4:**
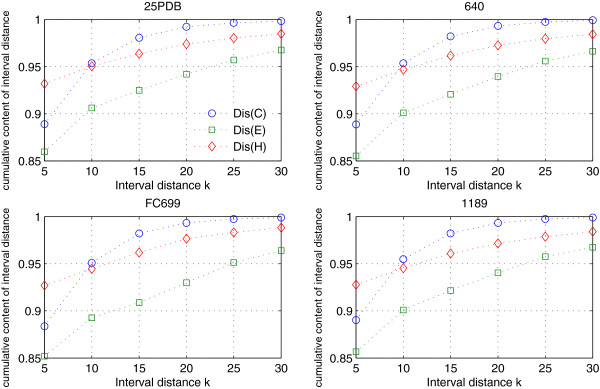
**The cumulative content of the interval distance for the datasets 25PDB, 640, FC699 and 1189.** The cumulative content of the interval distance for the datasets 25PDB, 640, FC699 and 1189. Here, we calculate the cumulative content of *Dis*(*C*), *Dis(E)* and *Dis*(*H*), and the interval distance is added up to *k*=5, 10, 15, 20, 25 and 30

From Figures 1 and 2, it is easy to find that more than 80% of the interval distances *Dis*(*δ*) are equal to 1, and the rest are too small. In order to show more clearly, we represented the cumulative content of the interval distances *Dis*(*δ*) for datasets 25PDB, 640, FC699 and 1189 in Figure 4. More details can be found in Additional file 2: Table S2. As would be expected, the content of the interval distances (*Dis*(*δ*) <5) is larger in four datasets, and their cumulative content of *Dis*(*δ*) <5for structure elements *C*, *E* and *H* are all well above 0.85. The cumulative content of the *Dis*(*δ*) increases from *k*=5 to *k*=30 for all four datasets. When *Dis*(*δ*) is equal to 30, all the cumulative content of the *Dis*(*δ*) are up to 0.96, especially for *Dis*(*C*) and *Dis*(*H*). That is to say, almost all the *Dis*(*δ*) are less than 30.

To show the influence of *k* parameter, we set the given interval distance k=5, 10, 15, 20, 25, 30 and calculated the *C*_(5)_(*δ*), *C*_(10)_(*δ*), *C*_(15)_(*δ*), *C*_(20)_(*δ*), *C*_(25)_(*δ*) and *C*_(30)_(*δ*) instead of *C*_*F*_(*δ*). We then evaluated their performance to discriminate the four major classes on datasets 25PDB, 640, FC699 and 1189, and their results are presented in Table 6.

**Table 6:** **Prediction accuracy of PBF-PSSE C**_**k**_**(δ) with selected parameter *k***

**Dataset**	**Method**	**Prediction accuracy (%)**				
		**All-α**	**All-β**	**α/β**	**α+β**	**Overall**
25PDB	*C*_(5)_(*δ*)	**76.98**	**80.14**	**63.87**	75.51	**74.72**
	*C*_(10)_(*δ*)	73.14	77.88	**63.87**	78	73.76
	*C*_(15)_(*δ*)	70.88	75.85	54.62	76.64	70.35
	*C*_(20)_(*δ*)	72.69	77.88	51.16	75.96	70.47
	*C*_(25)_(*δ*)	72.46	79.68	55.2	**77.78**	72.21
	*C*_(30)_(*δ*)	74.04	79.23	**63.87**	72.34	72.86
640	*C*_(5)_(*δ*)	**81.88**	75.97	**86.44**	**72.51**	**79.21**
	*C*_(10)_(*δ*)	77.54	81.82	82.49	71.93	78.44
	*C*_(15)_(*δ*)	76.81	**82.47**	67.80	62.57	71.86
	*C*_(20)_(*δ*)	78.26	78.57	72.88	67.84	74.06
	*C*_(25)_(*δ*)	78.99	79.87	82.49	64.08	76.25
	*C*_(30)_(*δ*)	78.26	74.68	83.62	66.08	75.63
FC699	*C*_(5)_(*δ*)	82.31	78.44	90.72	**64.63**	83.10
	*C*_(10)_(*δ*)	71.54	77.70	90.72	69.51	81.70
	*C*_(15)_(*δ*)	77.69	75.09	88.06	57.32	79.48
	*C*_(20)_(*δ*)	81.54	73.98	92.04	31.71	79.02
	*C*_(25)_(*δ*)	82.31	**78.81**	92.57	34.15	81.12
	*C*_(30)_(*δ*)	**84.62**	76.21	**93.63**	**64.63**	**84.03**
1189	*C*_(5)_(*δ*)	**77.58**	**86.39**	**85.03**	58.09	**77.93**
	*C*_(10)_(*δ*)	69.51	84.01	80.54	**59.75**	74.63
	*C*_(15)_(*δ*)	73.54	86.39	76.95	43.15	71.34
	*C*_(20)_(*δ*)	71.30	85.37	79.04	38.17	70.15
	*C*_(25)_(*δ*)	76.23	82.99	80.24	41.49	71.61
	*C*_(30)_(*δ*)	77.13	82.65	80.84	44.40	72.53

The accuracy of each class and overall accuracy of PBF-PSSE *C*_*k*_(*δ*) with selected parameter *k* for datasets 25PDB, 640, FC699 and 1189, and the parameter *k* is selected from the parameter set {5, 10, 15, 20, 25, 30}.

**Figure 5:**
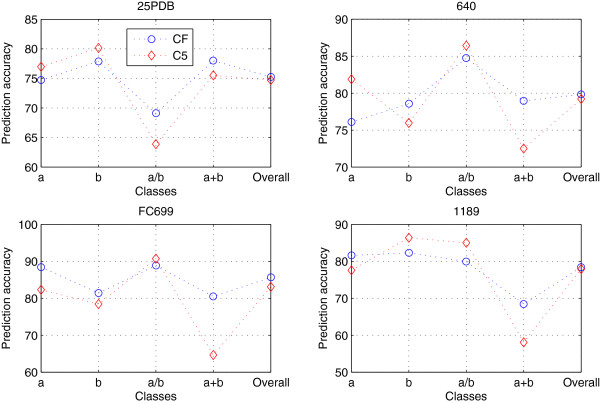
**Comparison of the position-based features *C***_***F***_**(*δ*) and *C***_**5**_**(*δ*) for the datasets 25PDB, 640, FC699 and 1189.** Comparison between *C*_*F*_(*δ*)and *C*_5_(*δ*) for the datasets 25PDB, 640, FC699 and 1189, where CF and C5 denote the PBF-PSSEs *C*_*F*_(*δ*) and *C*_5_(*δ*).

Table 6 largely confirms that the PBF-PSSE *C*_(*k*)_(*δ*) possess different performances based on different parameter *k*. The changes of the accuracy for the datasets 25PDB, 640, FC699 and 1189 are similar. The *C*_(5)_(*δ*) achieves the best performance among all *C*_(5)_(*δ*), *C*_(10)_(*δ*), *C*_(15)_(*δ*), *C*_(20)_(*δ*), *C*_(25)_(*δ*) and *C*_(30)_(*δ*). Figure 5 is the comparison of the overall accuracies between *C*_(5)_(*δ*) and *C*_(F)_(*δ*) for datasets 25PDB, 640, FC699 and 1189, more details can be found in Additional file 3: Table S3.

Take a closer look at Figure 5, we found that *C*_5_(*δ*) and *C*_*F*_(*δ*) have almost the similar performance. The overall accuracies of the *C*_5_(*δ*) for datasets 25PDB, 640, FC699 and 1189 are 74.72%, 79.21%, 83.10% and 77.93%, which are 0.53%, 0.63%, 2.56% and 0.46% lower than the *C*_*F*_(*δ*). These results are consistent with the cumulative content of the interval distance, so we can calculate the *C*_5_(*δ*) instead of the *C*_F_(*δ*), which can help you simplify equations in algebra, and also make some calculations easier.

## Conclusions

Prediction of structural classes for the low-homology datasets not only allows learning the overall folding type for a given protein sequence, but also helps in finding proteins that form similar folds in spite of low sequence similarity. Therefore, high quality prediction would be beneficial for in-silico prediction of tertiary structure of proteins with low sequence identity with respect to sequence used for prediction.

Numerous efficient methods have been proposed to predict protein structural classes for low-homology sequences, but challenge remains. In this paper, we aimed to develop a new method to improve prediction accuracy, which explores a potential way to capture the position information of predicted secondary structures. To do so, we first proposed a representation of the structure element position and analyzed the distance distribution of successive occurrences of an element, from which the semi-mean *Semi-E*_*(k)*_ and semi-variance *Semi-D*_(*k*)_ are calculated. Then, reciprocal of coefficient of variation was employed to construct the PBF-PSSE.

The main goal of our research is to investigate the importance of the PBF-PSSE and compare its performance with the CBF-PSSEs. The first contribution can be seen from the comparison with nine available CBF-PSSEs, we found that the PBF-PSSE is as important as the CBF-PSSEs, and *content*_*SE*_ are the most efficient CBF-PSSEs. The second contribution can be indicated from evaluation of the CBF-PSSEs combined with the PBF-PSSE, we noticed that the CBF-PSSEs’ prediction abilities are improved when combining with PBF-PSSE *C*_*F*_(*δ*), except for *MaxSeg*_*SE*_ and 3PATTERN. These results demonstrate that the PBF-PSSE and the CBF-PSSE have to work closely so as to make significant and complementary contributions to protein structural class prediction. The third contribution can be deduced from the performance of the proposed combined feature set PBF11CBF-PSSE and its comparison with competing prediction methods. Its overall accuracies for datasets 25PDB, 640, FC699 and 1189 are 86.25%, 86.41%, 94.99% and 84.71%, which are 1.91%, 2.97%, 5.39% and 1.51% higher than the existing best-performing method. The improvement can be contributed to the introduction of the PBF-PSSE that describes collocation of helix and strand segments in the predicted secondary structures. The final contribution can be seen from analysis of the influence of parameter *k*, we found that *C*_(*k*)_(*δ*) possesses different performances with different parameter *k, C*_5_(*δ*) and *C*_*F*_(*δ*) have almost the similar performance. So we can calculate the *C*_5_(*δ*) instead of the *C*_*F*_(*δ*) , which can help you simplify calculations.

Overall our comparison study highlights the necessity to extract more position information of the predicted secondary structures as possible. Thus, this understanding can be used to guide development of more powerful method for protein structural class prediction.

### Availability

Software name: PSCP-PSSE

Software home page: http://bioinfo.zstu.edu.cn/PSCP-PSSE

Operating system(s): windows

Programming languages: Matlab

License: web server freely available without registration

Restrictions to use by non-academics: on request

## Abbreviations

AA: Amino acid; APPA: Alternating frequency of *α*-helices and *β*-strands and proportion of parallel b-sheets and Anti-parallel b-sheets; AvgSegSE: Average length of the segment; CATH: Class, architecture, topology and homologous superfamily; CBF-PSSE: Content-based features of the predicted secondary structure elements; contentSE: Content of predicted secondary structure elements; CMV: Composition moment vector; CVPSSE: Coefficient of variability of predicted secondary structural elements; RCSB: Research collaboratory for structural bioinformatics; MaxSegSE: Length of the longest segment; NAvgSegSE: Normalized average length of the segment; NMaxSegSE: Normalized length of the longest segment; PBF-PSSE: Position-based features of the predicted secondary structure elements; PBF11CBF-PSSE: Combined PBF-PSSE with the 11-dimension CBF-PSSE Set; PSIBlast: Position-specific iterated BLAST; PSIPRED: Position specific iterated PRED; SCOP: Structural classification of proteins; SE: Secondary structure elements; SEFs: Sequence-based features; SeqLen: Length of the predicted secondary structure sequence; STFs: Structure-based features; SVM: Support vector machine; TPM: Transition probability matrix

## Competing interests

The authors declare that they have no competing interests.

## Authors’ contributions

QD conceived the method and prepared the manuscript. QD, LY and XQL implemented the software and performed the analysis. QD, YHY, YJC and PAH contributed to the discussion and have approved the final manuscript. All authors read and approved the final manuscript.

## Supplementary Material

Additional file 1: Table S1 The overall prediction accuracy for four data sets obtained with the CBF-PSSEs combined with the CBF-PSSE *content*_*SE*_. Click here for file

Additional file 2: Table S2 The cumulative content of the interval distance for the datasets 25PDB, 640, FC699 and 1189. Here, we calculate the cumulative content of *Dis*(*C*), *Dis*(*E*) and *Dis*(*H*), and the interval distance is added up to *k*=5, 10, 15, 20, 25 and 30. Click here for file

Additional file 3: Table S3 Comparison between PBF-PSSEs *C*_*F*_(*δ*) and *C*_5_(*δ*) for the datasets 25PDB, 640, FC699 and 1189, where CF and C5 denote PBF-PSSEs *C*_*F*_(*δ*) and *C*_*5*_(*δ*). Click here for file
