# Supplementary material for: Comparison study on statistical features of predicted secondary structures for protein structural class prediction: From content to position
Source: BMC Bioinformatics. 2013 May 4;14:152. doi: 10.1186/1471-2105-14-152 (PMC3652764; doi:10.1186/1471-2105-14-152)
Supplement: Additional file 1: Table S1 — The overall prediction accuracy for four data sets obtained with the CBF-PSSEs combined with the CBF-PSSE contentSE. [file 1471-2105-14-152-S1.doc]

STable 1. The overall prediction accuracies for four data sets obtained with the CBF-PSSEs combined with the CBF-PSSE$content_{SE}$.

| Methods | 25PDB | 640 | FC699 | 1189 |
| --- | --- | --- | --- | --- |
| $content_{SE}$+$C_{F}(\delta)$ | 83.14 | 85.78 | 94.87 | 84.43 |
| *CMV* +$content_{SE}$ | 75.79 | 80.47 | 88 | 78.75 |
| $MaxSeg_{SE}$+$content_{SE}$ | 67.3 | 67.34 | 83.57 | 70.51 |
| $NMaxSeg_{SE}$+$content_{SE}$ | 78.3 | 81.41 | 90.44 | 78.85 |
| $AvgSeg_{SE}$+$content_{SE}$ | 78.3 | 80.94 | 89.39 | 79.03 |
| $NAvgSeg_{SE}$+$content_{SE}$ | 74.66 | 79.22 | 85.90 | 78.11 |
| 3PATTERN +$content_{SE}$ | 67.78 | 66.72 | 83.68 | 67.77 |
| *APPA* +$content_{SE}$ | 77.17 | 81.09 | 86.83 | 81.5 |
| *TPM* +$content_{SE}$ | 77.05 | 79.69 | 88.69 | 81.04 |
