# Supplementary material for: Comparison study on statistical features of predicted secondary structures for protein structural class prediction: From content to position
Source: BMC Bioinformatics. 2013 May 4;14:152. doi: 10.1186/1471-2105-14-152 (PMC3652764; doi:10.1186/1471-2105-14-152)
Supplement: Additional file 2: Table S2 — The cumulative content of the interval distance for the datasets 25PDB, 640, FC699 and 1189. Here, we calculate the cumulative content of Dis(C), Dis(E) and Dis(H), and the interval distance is added up to k=5, 10, 15, 20, 25 and 30. [file 1471-2105-14-152-S2.doc]

S.Table 2. The cumulative content of the interval distance for the datasets 25PDB, 640, FC699 and 1189. Here, we calculate the cumulative content of $Dis(C)$, $Dis(E)$ and$Dis(H)$, and the interval distance is added up to k=5, 10, 15, 20, 25 and 30.

| Dataset | Method | Prediction accuracy (%) | | | | |
| --- | --- | --- | --- | --- | --- | --- |
|  |  | All-α | All-β | α/β | α+β | Overall |
|  | $C_{(5)}(\delta)$ | **0.7698** | **0.8014** | **0.6387** | 0.7551 | **0.7472** |
|  | $C_{(10)}(\delta)$ | 0.7314 | 0.7788 | **0.6387** | 0.78 | 0.7376 |
|  | $C_{(15)}(\delta)$ | 0.7088 | 0.7585 | 0.5462 | 0.7664 | 0.7035 |
|  | $C_{(20)}(\delta)$ | 0.7269 | 0.7788 | 0.5116 | 0.7596 | 0.7047 |
|  | $C_{(25)}(\delta)$ | 0.7246 | 0.7968 | 0.552 | **0.7778** | 0.7221 |
|  | $C_{(30)}(\delta)$ | 0.7404 | 0.7923 | **0.6387** | 0.7234 | 0.7286 |
| 640 | $C_{(5)}(\delta)$ | **0.913** | **0.7922** | **0.8814** | **0.7076** | **0.8203** |
|  | $C_{(10)}(\delta)$ | 0.8768 | 0.7792 | 0.8362 | 0.6491 | 0.7813 |
|  | $C_{(15)}(\delta)$ | 0.8913 | 0.7597 | 0.7062 | 0.5614 | 0.7203 |
|  | $C_{(20)}(\delta)$ | 0.8261 | 0.7143 | 0.7345 | 0.6316 | 0.7219 |
|  | $C_{(25)}(\delta)$ | 0.8188 | 0.7078 | 0.8362 | 0.5906 | 0.7359 |
|  | $C_{(30)}(\delta)$ | 0.8043 | 0.7273 | 0.8362 | 0.5731 | 0.7328 |
| FC699 | $C_{(5)}(\delta)$ | 0.9538 | **0.9145** | **0.9284** | 0.5244 | **0.8893** |
|  | $C_{(10)}(\delta)$ | **0.9615** | **0.9145** | 0.9151 | 0.5122 | 0.8834 |
|  | $C_{(15)}(\delta)$ | **0.9615** | 0.8699 | 0.8806 | 0.4756 | 0.8508 |
|  | $C_{(20)}(\delta)$ | 0.8769 | 0.8364 | 0.9231 | 0.2317 | 0.8228 |
|  | $C_{(25)}(\delta)$ | 0.8385 | 0.881 | 0.9125 | 0.3293 | 0.8357 |
|  | $C_{(30)}(\delta)$ | 0.8692 | 0.8662 | 0.931 | **0.5488** | 0.8648 |
| 1189 | $C_{(5)}(\delta)$ | **0.9372** | **0.8469** | **0.8263** | 0.5643 | **0.7967** |
|  | $C_{(10)}(\delta)$ | 0.9193 | 0.8639 | 0.7605 | **0.5934** | 0.7839 |
|  | $C_{(15)}(\delta)$ | 0.8924 | 0.8503 | 0.7485 | 0.3942 | 0.7271 |
|  | $C_{(20)}(\delta)$ | 0.8655 | 0.8367 | 0.7695 | 0.361 | 0.717 |
|  | $C_{(25)}(\delta)$ | 0.8206 | 0.7993 | 0.7874 | 0.4315 | 0.7189 |
|  | $C_{(30)}(\delta)$ | 0.7982 | 0.7789 | 0.7904 | 0.4896 | 0.7225 |
