# Supplementary material for: Comparison study on statistical features of predicted secondary structures for protein structural class prediction: From content to position
Source: BMC Bioinformatics. 2013 May 4;14:152. doi: 10.1186/1471-2105-14-152 (PMC3652764; doi:10.1186/1471-2105-14-152)
Supplement: Additional file 3: Table S3 — Comparison between PBF-PSSEs CF(δ) and C5(δ) for the datasets 25PDB, 640, FC699 and 1189, where CF and C5 denote PBF-PSSEs CF(δ) and C5(δ). [file 1471-2105-14-152-S3.doc]

S.Table 3. Comparison between PBF-PSSEs $C_{F}(\delta)$ and $C_{5}(\delta)$ for the datasets 25PDB, 640, FC699 and 1189, where CF and C5 denote PBF-PSSEs $C_{F}(\delta)$ and $C_{5}(\delta)$.

| Datasets | Methods | All-α | All-β | α/β | α+β | Overall |
| --- | --- | --- | --- | --- | --- | --- |
| 25PDB | CF | 74.72 | 77.88 | 69.08 | 78.00 | 75.25 |
|  | C5 | 76.98 | 80.14 | 63.87 | 75.51 | 74.72 |
| 640 | CF | 76.09 | 78.57 | 84.75 | 78.95 | 79.84 |
|  | C5 | 81.88 | 75.97 | 86.44 | 72.51 | 79.21 |
| FC699 | CF | 88.46 | 81.41 | 88.86 | 80.49 | 85.66 |
|  | C5 | 82.31 | 78.44 | 90.72 | **64.63** | 83.10 |
| 1189 | CF | 81.61 | 82.31 | 79.94 | 68.46 | 78.39 |
|  | C5 | 77.58 | 86.39 | 85.03 | 58.09 | 77.93 |
